# Integrative Analysis Reveals the Diverse Effects of 3D Stiffness upon Stem Cell Fate

**DOI:** 10.3390/ijms24119311

**Published:** 2023-05-26

**Authors:** Muxin Yue, Yunsong Liu, Ping Zhang, Zheng Li, Yongsheng Zhou

**Affiliations:** 1 Department of Prosthodontics, Peking University School and Hospital of Stomatology, Beijing 100081, China; yuemuxin@hsc.pku.edu.cn (M.Y.); liuyunsong@hsc.pku.edu.cn (Y.L.); zhangping332@hsc.pku.edu.cn (P.Z.); 2 National Center for Stomatology & National Clinical Research Center for Oral Diseases & National Engineering Research Center of Oral Biomaterials and Digital Medical Devices & Beijing Key Laboratory of Digital Stomatology, Beijing 100081, China

**Keywords:** extracellular matrix, mesenchymal stem cells, stiffness, metabolism

## Abstract

The origin of life and native tissue development are dependent on the heterogeneity of pluripotent stem cells. Bone marrow mesenchymal stem cells (BMMSCs) are located in a complicated niche with variable matrix stiffnesses, resulting in divergent stem cell fates. However, how stiffness drives stem cell fate remains unknown. For this study, we performed whole-gene transcriptomics and precise untargeted metabolomics sequencing to elucidate the complex interaction network of stem cell transcriptional and metabolic signals in extracellular matrices (ECMs) with different stiffnesses, and we propose a potential mechanism involved in stem cell fate decision. In a stiff (39~45 kPa) ECM, biosynthesis of aminoacyl-tRNA was up-regulated, and increased osteogenesis was also observed. In a soft (7~10 kPa) ECM, biosynthesis of unsaturated fatty acids and deposition of glycosaminoglycans were increased, accompanied by enhanced adipogenic/chondrogenic differentiation of BMMSCs. In addition, a panel of genes responding to the stiffness of the ECM were validated in vitro, mapping out the key signaling network that regulates stem cells’ fate decisions. This finding of “stiffness-dependent manipulation of stem cell fate” provides a novel molecular biological basis for development of potential therapeutic targets within tissue engineering, from both a cellular metabolic and a biomechanical perspective.

## 1. Introduction

The heterogeneity of pluripotent stem cells is the basis of tissue development. Mesenchymal stem cells (MSCs) are located within a three-dimensional (3D) microenvironment that comprises multiple components [1,2]. It has been reported that the physical characteristics of the extracellular matrix (ECM) affect cellular behaviors by both regulating developmental processes and tissue homeostasis [3,4]. Stiffness is one of the most significant physical properties that cells can recognize [5], and in different tissues or microenvironments stiffness dynamically changes spatiotemporal gene expression.

Numerous studies have shown that stiffness plays a vital role in regulating stem cell fate [6], and mechanistic studies have identified that mechano-transduction pathways in cells can sense and translate stiffness signals into intracellular signaling pathways, which results in broad gene expression changes [7].

Under two-dimensional (2D) culture conditions, numerous recent studies have shown that ECM stiffness can induce MSCs to differentiate into nerve, cartilage, muscle, and bone. Engler et al. [8] first studied the regulation effects of ECM stiffness on MSCs at the 2D level. This study demonstrated that when MSCs were cultured in a matrix of between 0.1 and 1.0 kPa (simulating the stiffness of brain tissue), MSCs showed the branching morphology of nerve cells. When MSCs were cultured in a matrix of between 8 and 17 kPa (simulating muscle tissue stiffness), MSCs became spindle-like myoblasts. Finally, in a matrix cultured between 25 and 40 kPa (simulating bone-like stiffness), MSCs showed osteoblast-like morphology [8]. Further studies capturing varied transcriptional responses to different ECM stiffnesses in 2D models have reinforced these findings [9,10]. For example, it was found that the expression of osteogenesis-related markers was up-regulated in a stiffer ECM, and the expression of adipogenesis-related markers were down-regulated in a less rigid ECM [11]. Moreover, it was shown that MSCs could respond to ECM stiffness through NFκB-p65 activity in the stiffness-dependent pathway [7]. However, caution should be exercised as the behaviors of stem cells under 2D culture conditions are different than those within a natural 3D ECM due to complicated mechano-transduction and force generation effects within 3D contexts [12]. The effect of ECM stiffness upon the differentiation of MSCs under 3D culture is similar to the previous studies under 2D culture [10,13,14,15,16,17].

Pek et al. [13] applied liquefaction shear strength (τ_y_) to gels with stiffnesses of 7, 25, 40, 75, and 100 Pa. They found that when the stiffness of the ECM was 7 Pa, the expression of neuro-marker ENO2 was up-regulated, while the myogenic marker molecule MYOD and the osteogenic marker molecule RUNX2 were up-regulated, respectively, in the medium stiffness matrix (τ_y_ = 25 Pa) and higher stiffness matrix (τ_y_ = 75 and 100 Pa). These findings indicate that stem cells can “feel” and interpret changes to a specific stiffness, leading to differentiations in marker expression. However, exactly how the mechanical cues of ECM stiffness are converted into intracellular biochemical signals to regulate a MSC’s fate still needs systematic and in-depth exploration. One study by Baek et al. [18] revealed that different mechanistic processes could lead to varied mechanosensitive lineage commitments in a 3D ECM compared with a 2D ECM. In addition, the unbiased RNA-seq showed that a stiff ECM could improve the expression of neuro-markers, including early growth response 1 (Egr1) and neural cell adhesion molecule 1 (Ncam1).

Mechanistic studies have identified that mechano-transduction pathways in cells can sense and translate stiffness signal into intracellular signaling pathways, which brings about broad gene expression changes [7]. Identification of how specific cues in the microenvironment control stem cell fate will provide potential strategies for tissue engineering [19]. Previously, there were some high-throughput studies on the regulation of stem cell fate according to ECM stiffness, but most of them were single high-throughput studies [7,8,9]. More recently, the influence of metabolic cues induced by ECM stiffness determining stem cell fate have been investigated [20,21]. For example, alterations in ECM stiffness affect glucose, lipid, and amino acid metabolism, thereby altering cell phenotype and regulation of cell fate [22]. Manipulation of stiffness on metabolism can be orchestrated using multiple pathways, including the Yes-associated protein and transcriptional co-activator with the PDZ-binding motif (YAP/TAZ) pathway, the thioredoxin-interacting protein (TXNIP) pathway, the Ras homolog family and Rho-associated coiled coil containing protein serine/threonine kinase (Rho/ROCK) pathways, the integrin-focal adhesion kinase- phosphatidylinositol 3-kinase-protein kinase B (integrin-FAK-PI3K-Akt) pathway, and the AMP-activated protein kinase (AMPK) pathway [23]. However, most studies of metabolic changes responding to ECM stiffness focused only on the phenomenon, and very few metabolites and precise metabolic pathways have been elucidated [23,24].

An interesting study revealed that under soft 2D ECM conditions, the metabolites of glycolysis and the tricarboxylic acid cycle were systematically down-regulated [25]. However, metabolomic sequencing only displayed the metabolic profiles of metabolites responding to 2D stiffness and did not provide evidence of stem cell fate decision and differentially expressed pathways. The lack of a systematic multi-omics analysis of metabolic processes influenced by 2D or 3D ECM stiffness hinders the in vivo implications for stiffness in regulation of stem cell fate.

**Figure 1:**
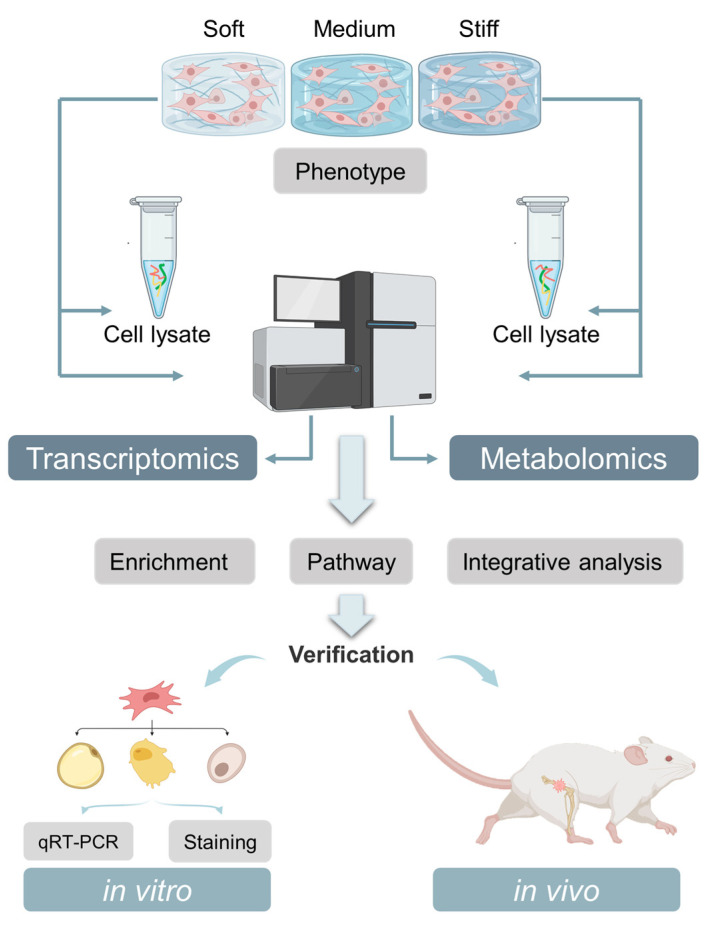
A schematic illustration of multi-chip high-throughput mapping analysis demonstrating how ECM stiffness regulates the fate of BMMSCs.

Biological processes during the regulation of stem cell fate are complicated, and it is difficult to comprehensively understand the regulatory mechanism of this intricated physiological process from single-cell omics [7]. In this study, we systematically investigated the gene and metabolic profiles, as well as the stem cell fates, in ECMs with different stiffnesses. Transcriptomic and metabolomic results were analyzed in combination. Key genes, metabolites, and metabolic pathways were filtered to comprehensively explain the regulation of stem cell fate (Figure 1). Furthermore, we conducted in vitro and in vivo experiments in a 3D ECM to verify the vital genes and pathways within the stem cell fate decision process that directly respond to stiffness changes. Overall, we provide new ideas for understanding the regulation effect of ECM stiffness in altering stem cell fate from the metabolic viewpoint.

## 2. Results and Discussion

### 2.1. Characterization of GelMA Stiffness

GelMA hydrogels have excellent biocompatibility and provide a good 3D structure for cell proliferation and differentiation (Figure S1A,B). Stiffness of the hydrogels was adjusted by changing the matrix formulation. Three hydrogels were prepared: GM30, GM60, and GM90, with GM30 being the least stiff and GM90 the most stiff. The compression modulus of the soft group (GM30) was determined to be 9.19 ± 1.68 kPa, the medium group (GM60) was 25.46 ± 4.96 kPa, and the stiff group (GM90) was 42.16 ± 3.29 kPa (Figure S1C,D). These hydrogels therefore provide a favorable platform for investigation of the effect of ECM stiffness on stem cell fate.

### 2.2. Overview of Transcriptome Analysis

**Figure 2:**
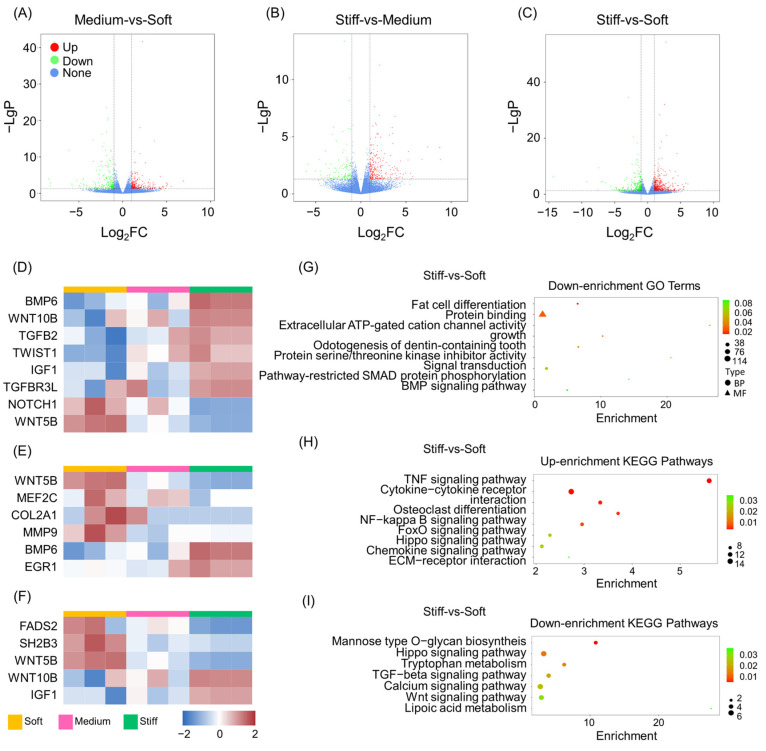
Transcriptome changes in BMMSCs cultured in ECMs with different stiffnesses after 7 days. (**A**–**C**) Volcano plots of the DEGs based on both magnitude of the *p* value and fold changes (FCs) in (**A**) medium group compared with soft group, (**B**) stiff group compared with medium group, and (**C**) stiff group compared with soft group. (**D**–**F**) Heatmap of typical DEGs associated with (**D**) osteogenesis, (**E**) chondrogenesis, and (**F**) adipogenesis between MSCs cultured in ECMs with three different stiffnesses. K-means clusters are indexed using colored bars. (**G**) GO enrichment analysis of the down-regulated genes in stiff-vs-soft groups. (**H**) KEGG pathway analysis of up-regulated genes in stiff-vs-soft groups. (**I**) KEGG pathway analysis of down-regulated genes in stiff-vs-soft groups.

RNA-seq based on next-generation sequencing technology was used to explore global gene expression changes in stem cell fate [7]. BMMSCs were cultured in vitro using GelMA scaffolds with three different stiffnesses (GM30, GM60, and GM90), and after 7 days RNA-seq was performed. The results showed that a large number of genes were up- or down-regulated between each group, with stiffness having an impact. Differences between expressed genes (DEGs) were interrogated using volcano plots (Figure 2A–C). As a result, 977 DEGs were obtained between the soft and stiff groups, 764 DEGs between the soft and medium groups, and 594 DEGs between the medium and stiff groups. There were also similarities found between the three biological replicates of BMMSCs with different stiffnesses cultured in the ECM. All three replicates were further analyzed to prove that the sequencing results were consistent.

The results showed that with the change of stiffness, cells in 3D culture presented different trends, with osteogenic, adipogenic, and chondrogenic differentiation observed. In general, elevation of ECM stiffness enhanced the osteogenic ability of BMMSCs, while the chondrogenic and adipogenic differentiation of BMMSCs was diminished. Specifically, there was an increase in DEGs observed relating to osteogenesis (Figure 2D) such as those with bone morphogenetic protein 6 (BMP6), Wnt family member 10B (WNT10B), TGFB2, twist family basic helix–loop–helix transcription factor 1 (TWIST1), insulin-like growth factor 1 (IGF1), and transforming growth factor beta receptor 3-like (TGFBR3L). Conversely, notch receptor 1 (NOTCH1) and Wnt family member 5B (WNT5B) exhibited the opposite trend. Recently, high stiffness has been found to activate the Wnt signaling pathway, which regulates osteogenesis through mechano-transduction [26]. However, the functions of the Wnt family were varied [27]. An in vivo study revealed that the bone mineral density (BMD) of mice was reduced with high-throughput knockdown of *Wnt10b*, but the BMD of mice increased in knockdown of *Wnt5b* [28].

Conversely, when the stiffness of the matrix was low, expression of chondrogenic-related genes (Figure 2E) including WNT5B, myocyte enhancer factor 2C (MEF2C), COL2A1, and matrix metallopeptidase 9 (MMP9) were also increased, but the gene expression of BMP6 and early growth response 1 (EGR1) decreased. The latest study has shown that with different ECM stiffnesses, BMP family members exhibit different biological activities, which in turn have an impact on skeletal progenitor cell adhesion and differentiation [29]. BMP6 expression improved bone formation and inhibited osteoclast differentiation in osteoporotic rats, therefore uncoupling from bone remodeling. Compared with BMMSCs, adipose-tissue-derived stem cells (ASCs) showed an increased chondrogenic potential, especially with BMP-6 treatment [30]. Moreover, BMP6 can modulate glucose and iron metabolism, which suggests that it plays an important role in metabolic regulation [31,32]. Finally, Palomares et al. [33] found that a mechanical bending motion enhanced the expression of genes related to chondrogenesis, such as COL2A1, and repressed expression of genes related to osteogenesis, such as BMP6. It can therefore be inferred that both BMP and collagen family members are key factors in the selective regulation of bone formation and cartilage formation via mechanical signaling.

Genes relating to adipogenesis showed differences responding to different stiffnesses (Figure 2F). Interestingly, fatty acid desaturase 2 (FADS2), SH2B adaptor protein 3 (SH2B3), the lymphocyte adaptor protein (LNK1), and WNT5B were extensively expressed in the soft ECM samples, whereas WNT10B and IGF1 displayed the opposite trend. FADS2 is a key fatty acid desaturase involved in regulating cellular lipid metabolic activities [34]; SH2B3 is also known as the lymphocyte adaptor protein (LNK1), with the SH2B family responsible for regulation of the insulin/IGF-1-receptor-related pathway for adipogenesis both in vivo and in vitro [35,36]; and IGF1 is an autocrine factor that promotes cell-to-cell communication and cell division. It also participates in the negative regulation of adipogenesis [37,38]. As discussed above, WNT5B plays a negative role in osteogenesis [39], and a positive role in chondrogenesis and adipogenesis. WNT5B also regulates adipogenic differentiation by increasing the level of adipogenesis-related markers and promotes adipogenesis by inhibiting nuclear translocation of β-catenin and repressing typical WNT target genes, such as IGF-1 [40,41]. Our results indicated a similar trend; in a soft ECM the expression of WNT5B increased, while IGF1 decreased, thus leading to enhancement of adipogenesis.

### 2.3. GO and KEGG Pathway Analysis of DEGs

Although RNA-seq showed that gene expression changed with stiffness, the biological functions and relationships between DEGs remains unclear. GO enrichment analysis was carried out to assess the function of DEGs in stem cells cultured in ECMs with different stiffnesses. The results showed that compared with soft group, inflammatory response, cell–cell signaling, cellular response to hypoxia, positive regulation of gene expression, positive regulation of ossification, modulation of cell proliferation, extracellular space, and extracellular matrix protein binding were all up-regulated in the stiff group (Figure S2). Meanwhile, fat cell differentiation, BMP signaling pathways, protein binding, and signal transduction were down-regulated (Figure 2G). GO enrichment comparisons between the stiff and medium groups and the medium and soft groups showed similar results (Figures S3A,B and S4A,B). Compared with the soft group, regulation of cell proliferation, osteoblast differentiation, and cell growth via extracellular and glucose metabolic processes were enhanced in the medium group, but the steroid biosynthetic process and fat cell differentiation were diminished. Regulation of actin cytoskeleton organization was enhanced in the medium group, and regulation of cartilage development, bone resorption, chondrocyte differentiation, and fat cell differentiation were reduced.

KEGG pathway enrichment analysis was conducted to explore signaling pathways that influence the DEGs between ECMs with different stiffnesses. Compared with the soft group, the TNF signaling pathway, cytokine–cytokine receptor interaction, osteoclast differentiation, the NF-κB signaling pathway, the FoxO signaling pathway, the Hippo signaling pathway, the chemokine signaling pathway, and ECM-receptor interactions were all up-regulated in the stiff group (Figure 2H). Meanwhile, mannose-type *O*-glycan biosynthesis, tryptophan metabolism, the TGF-β signaling pathway, the calcium signaling pathway, the Wnt signaling pathway, and lipoic acid metabolism were all down-regulated in the stiff group (Figure 2I). In the medium group, the FoxO, HIF-1, and TGF-β signaling pathways all revealed up-regulation when compared to the stiff group, while lipoic acid metabolism and steroid hormone biosynthesis were down-regulated. (Figure S3C,D). In addition, compared to the soft group, expression of TNF and Rap1 were enhanced, but the pathways for calcium signaling, Wnt signaling, and cAMP signaling showed a decrease in expression (Figure S4C,D).

The Hippo signaling pathway is a key process in mechanobiology and plays a significant role in bone homeostasis [42]. YAP and TAZ are the main downstream regulators of the Hippo signaling pathway [43], with YAP/TAZ being an important central effector of mechanosensory pathways within MSCs that can translate mechanical signals into biochemical signals [44]. Knockout of Yap enhances osteogenesis and inhibits adipogenesis via the β-catenin signaling pathway, which is evidence that YAP is important for the Wnt/β-catenin signaling pathway [45]. The results of both GO and KEGG pathway analysis demonstrated that increased ECM stiffness is transmitted to downstream signaling pathways through activation of mechano-transduction pathways, thus promoting osteogenic differentiation while inhibiting adipogenesis.

### 2.4. Overview of Metabolomics Analysis

**Figure 3:**
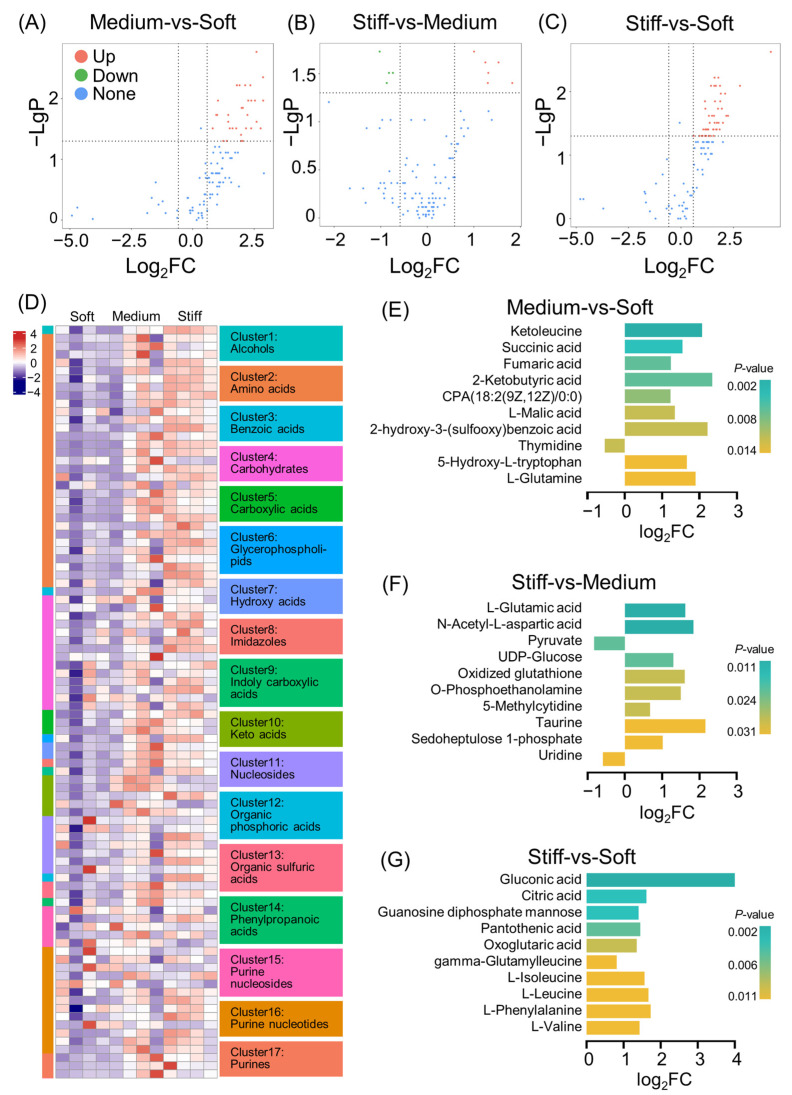
Overview of metabolomics analysis between each group of ECMs for 7 days. (**A**–**C**) The metabolites that were the most significantly different in each pairwise comparison between two groups. Dunn’s tests were used for pairwise comparisons; *p* < 0.05 was considered statistically significant. (**D**) Heatmap showed differences in metabolites using log2-transformed fold changes. K-means clusters are indexed using colored bars. (**E**–**G**) Significantly differential metabolites between (**E**) medium and soft groups, (**F**) stiff and medium groups, and (**G**) stiff and soft groups. *p* value is displayed using colored bars.

Metabolomics analysis, using high-performance liquid chromatography in tandem with mass spectrometry (HPLC–MS), detected 21 classes of metabolite including amino acids, carbohydrates, and purine nucleotides [46]. Compared with the soft group, 31 kinds of metabolites increased in the medium group (Figure 3A), and 45 were enhanced in the stiff group (Figure 3B). In addition, when compared with the medium group, six types of metabolites were up-regulated, but four were reduced in the stiff group (Figure 3C). A global clustering heatmap showed metabolites that were significantly changed in BMMSCs cultured in an ECM when stiffness was taken into account (Figure 3D). The possible reason for these differences may be that increased ECM stiffness leads to enhanced cellular biological activity, which needs more energy to maintain intracellular homeostasis and thus changes the cell metabolism [47,48]. However, the prospective mechanism of this phenomenon remains to be further investigated.

Significantly different metabolites were plotted based on the sequencing results, which were demonstrated via the fold change of the top metabolites and *p* values (Figure 3E–G). When compared with the soft group, up-regulated top metabolites in the medium group included ketoleucine, succinic acid, fumaric acid, 2-ketobutyric acid, CPA (18:2(9-Z,12-Z)/0:0), L-malic acid, 2-hydroxy-3-(sulfoxy) benzoic acid, 5-hydroxy-L-tryptophan, and L-glutamine. On the other hand, the presence of thymidine was reduced when the medium and soft groups were compared (Figure 3E).

In the stiff group, the results revealed that up-regulated top metabolites included L-glutamic acid, *N*-acetyl-L-aspartic acid, UDP-glucose, oxidized glutathione, *O*-phosphoethanolamine, 5-methylcytidine, taurine, and sedoheptulose 1-phosphate. Both pyruvate and uridine were down-regulated in the stiff group when compared to the medium group (Figure 3F). Comparison of the stiff group with the soft group revealed that gluconic acid, citric acid, guanosine diphosphate mannose, pantothenic acid, oxoglutaric acid, gamma-glutamyl leucine, L-isoleucine, L-lleucine, L-phenylalanine, and L-valine were all up-regulated top metabolites (Figure 3G). Our results indicated that most of these metabolites were related to glucose metabolism (Figure 3E–G). This aligns with other investigations, where it has been reported that a high stiffness of ECM-promoted cell proliferation and osteogenesis of MSCs were both observed due to enhanced ATP levels and AMPK activation [22], as well as increased glycolysis [5,49], mitochondrial production [50], and processes up-regulating the uptake and utilization of glucose.

ECM stiffness is associated with glutamine catabolism through direct activation of glutaminase transcription mediated by the YAP-TEAD pathway [51,52]. Glutamine is the precursor for the synthesis of proteins, nucleic acids, and many other biomolecules, and the metabolites of glutamine can be involved in the tricarboxylic acid (TCA) cycle. ECM stiffening activates glycolysis and glutamine metabolism, thereby coordinating the cycling of non-essential amino acids in the cellular microenvironment [52]. A recent study found that ECM stiffening reprogrammed glutamine metabolism in breast cancer cells, which promoted microtubule glutamylation and forced microtubule stabilization, thereby facilitating cell invasion [53]. Glutamine-derived α-ketoglutarate was found to support amino acid biosynthesis and proliferation in skeletal stem cells, whereas inhibition of glutamine metabolism resulted in reduced bone mass and increased bone marrow adiposity in mice [54].

ECM stiffness also regulates amino acid transport through regulation of the YAP/TAZ pathway [52]. Bone remodeling is an energy-consuming process, and amino acids are an alternative source of energy [55,56]. In our study, expression of the branched amino acids L-isoleucine, L-leucine, and L-valine were elevated in the stiff ECMs, which is shown in Figure 3G. In conclusion, analysis of metabolites suggests that the metabolites themselves play an important role in regulation of stem cell differentiation in ECMs with different stiffnesses.

### 2.5. Metabolite Set Enrichment Analysis and Metabolic Pathway Analysis

**Figure 4:**
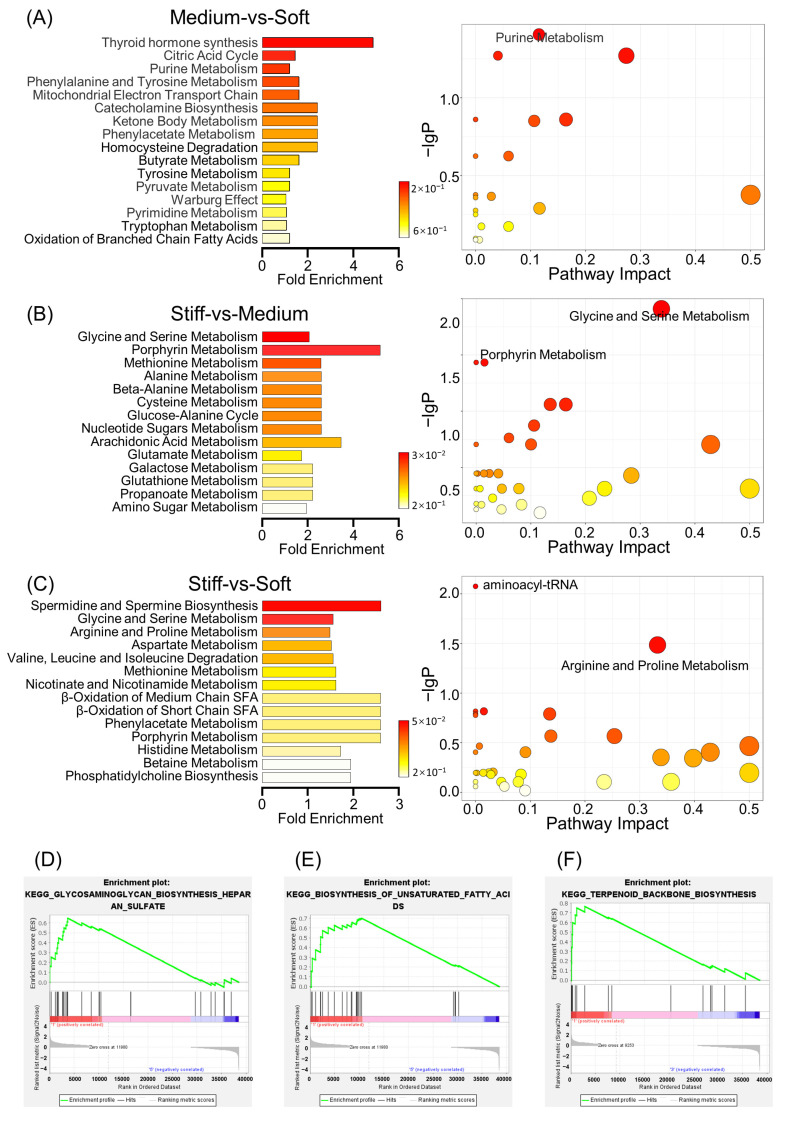
Metabolite Set Enrichment Analysis and Metabolic Pathway Analysis in relation to ECM stiffness. (**A**–**C**) Lift line: The overview of metabolite set enrichment between each pairwise comparison on ECM stiffness. Right line: Metabolic pathway analysis about ECM stiffness. *p* value was displayed by color bar. (**D**–**F**) GSEA was performed to identify the comprehensive analysis of metabolomics and transcriptomics.

Metabolite set enrichment analysis (MSEA) has gained growing interest for identification of metabolic pathways in metabolomics [57]. Comparison between the medium group and soft group showed that the key differential metabolites included those from thyroid hormone synthesis, the citric acid cycle, purine metabolism, and the mitochondrial electron transport chain, among others (Figure 4A). Meanwhile, the fold changes reached 4.88, 1.46, 1.22, and 1.63, respectively. The 18 metabolites (*p* < 0.05) used for metabolic pathway analysis presented significant differences, particularly for those generated during pyruvate metabolism. Comparing the stiff group with the medium group, the metabolites arising from glycine and serine metabolism, porphyrin metabolism, and methionine metabolism were significantly different (Figure 4B). Comparison of MSEA between the stiff and soft groups displayed that both spermidine and spermine biosynthesis and glycine and serine metabolites were the most significant. The spermidine and spermine biosynthesis, arginine and proline metabolism, and aminoacyl-tRNA biosynthesis pathways all demonstrated correlation with ECM stiffness, with a *p* value less than 0.05, indicating statistical significance (Figure 4C).

It has been reported that inhibition of spermidine synthase in MSCs leads to diminished bone formation in vitro, which can be verified by decreased mineralization and bone sialoprotein expression [58]. Spermidine synthase-deficient MSCs showed high levels of mitochondrial fusion, deterioration of mitochondrial function, reduced glucose consumption, and increased lactate secretion. Gene information is translated into functional proteins via effective transcription and translation [59]. For this to occur, mechanical signals are propagated in the nucleus for regulation of the state of chromatin and transcription, where the synthesis of aminoacyl-tRNA plays a critical role [48]. In a recent combined proteomic and metabolomic analysis relating to osteogenic differentiation of dental pulp stem cells, a four-fold increase in protein levels of aminoacyl-tRNA synthetase was found [60]. Consistently, our data suggest that osteogenic differentiation was enhanced in stiff ECMs and that aminoacyl-tRNA synthesis was increased, which indicates that aminoacyl-tRNA may be related to induced osteogenesis in stiff ECMs.

Current studies suggest that both cytoskeletal and mechanical factors commonly affect the structure and function of mitochondrial networks [61]. However, studies have not deeply investigated the underlying mechanism for how stiffness affects cell differentiation by influencing mitochondrial function. ATP, as the primary energy source for supporting almost all cellular activities, is derived from the electron transport chain [62]. Mitochondrial dysfunction would impair osteogenesis and accelerate age-related bone loss [63]. Li et al. showed that, in particular, the mitochondrial membrane potential, NAD+/NADH ratio, and ATP production of BMMSCs in aged mice were lower than those in young mice [64]. During our MSEA studies, it was shown that the mitochondrial electron transport chain, which is up-regulated with increased stiffness, promotes ATP production and thus provides more energy for cell differentiation (Figure 4A–C). In summary, we observed that metabolic pathways were greatly altered in ECMs with different stiffnesses, with the possible reason being different demands for cell differentiation.

### 2.6. Comprehensive Analysis of Metabolomics and Transcriptomics

In order to identify the functional enrichment of different ECM stiffnesses, gene set enrichment analysis (GSEA) was performed. The KEGG enrichment term showed that the stiffness of the ECM was mainly associated with biosynthesis-related activities. In a soft ECM, incidences of the glycosaminoglycan biosynthesis of heparan sulfate and the biosynthesis of unsaturated fatty acid increased (Figure 4D,E); these are both important processes for chondrogenic differentiation and adipogenic differentiation of cells, respectively. Glycosaminoglycan has a wide range of effects upon cancer initiation and progression, including regulating cellular metabolism, acting as a sensor for mechanical properties of ECMs, and playing part in therapeutic resistance [65,66]. A possible reason for this may be that glycosaminoglycan could serve as a molecular bridge between the ECM and cells, in a cell-specific and context-specific manner.

Finally, it was noted that the identity of any terpenoid metabolite was altered as the stiffness changed (Figure 4F). Terpenoids are a family of natural products that have attracted wide attention because of their various biological activities. They are associated with the biosynthesis of cholesterol in microglia, which are involved in lipid metabolism [67]. It has been found that terpenoids play a role in the treatment of osteoporosis by promoting bone deposition and inhibiting bone resorption [68], but the specific mechanism of action needs further investigation.

### 2.7. Validation In Vitro and In Vivo

**Figure 5:**
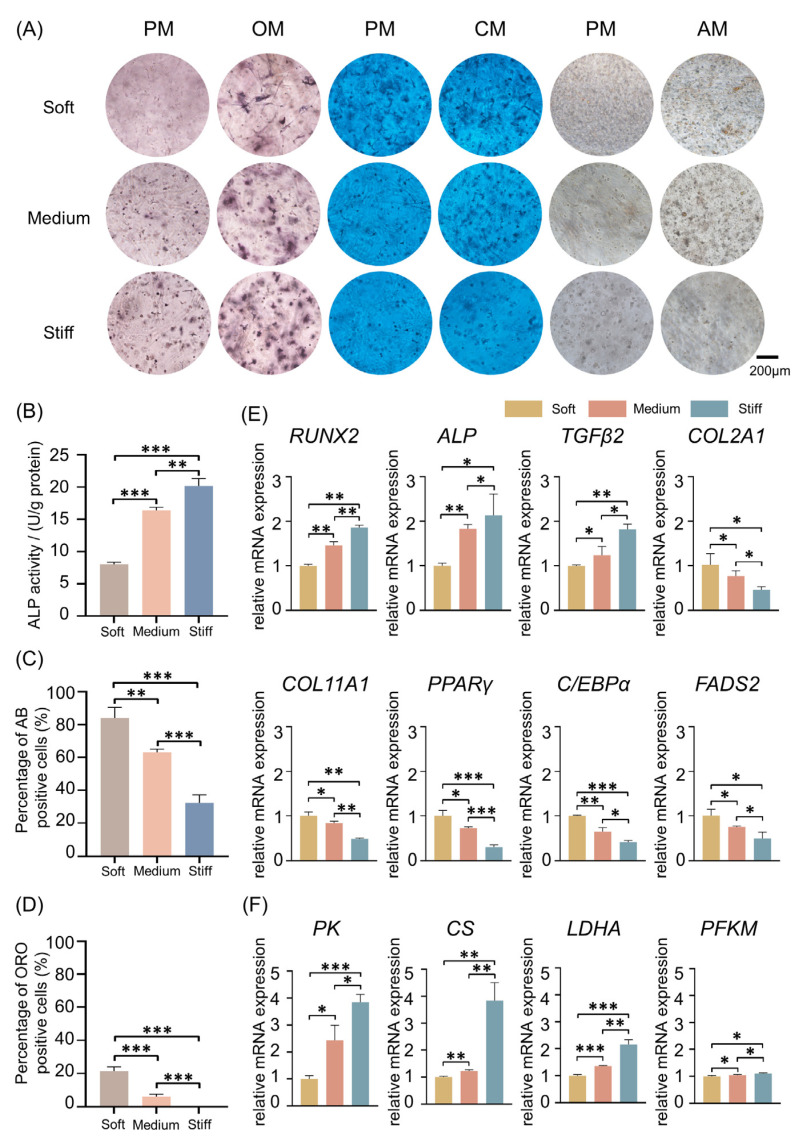
Verification the effect of ECM stiffness on stem cell fate in vitro. (**A**) ALP staining (7 days), Alcian blue staining (14 days), and Oil Red O staining (21 days) in soft, medium, and stiff groups. Scale bar is 200 μm. (**B**) ALP activity in soft, medium, and stiff groups. (**C**,**D**) Quantification of the percentage of positive staining cells in Alcian blue (**C**) and Oil Red O (**D**) in soft, medium, and stiff groups. (**E**) The qRT-PCR analysis showed genes related to stem cell fate in soft, medium, and stiff groups (*RUNX2, ALP, TGFβ2, COL2A1*, and *COL11A1* for 7 days; *PPARγ*, *C/EBPα*, and *FADS2* for 14 days). (**F**) The qRT-PCR analysis showed genes related to cell metabolism in soft, medium, and stiff groups for 7 days. (* *p* < 0.05, ** *p* < 0.01, *** *p* < 0.001, determined using one-way ANOVA).

To verify the role of different ECM stiffnesses on stem cell fate, ALP staining, Alcian blue staining, and Oil Red O staining were all performed to test osteogenic, chondrogenic, and adipogenic differentiation of BMMSCs in soft, medium, and stiff ECMs (Figure 5A). After 7 days of cell 3D culture in proliferation medium (PM) and osteogenic medium (OM), ALP staining and activity indicated that, with the elevation of ECM stiffness, osteogenic differentiation was enhanced (Figure 5B). However, after 14 days, Alcian blue staining suggested that chondrogenic differentiation of MSCs was the highest in the soft group, followed by the medium group and then the stiff group. After being cultured in PM and adipogenic medium (AM) for 21 days, Oil Red O staining was applied to verify the role of ECM stiffness upon adipogenic differentiation. The results showed that more fat droplets were formed in the soft group than in the medium group. In the stiff group, there was almost no fat droplet formation. The percentages of positive cells when stained with either Alcian blue or Oil Red O were calculated and were consistent with the result of staining (Figure 5C,D). Numerous studies have reported that a soft ECM leads to adipogenesis and chondrogenesis of MSCs, and a stiff ECM contributes to osteogenesis, which is in accordance with our results [69,70]. However, for chondrogenic differentiation the picture is more complex; it is not only stiffness that determines the tendency of chondrogenesis. A recent study adjusted the weight ratio of poly (ethylene glycol) diacrylate (PEGDA) and a concentration of GelMA, revealing that a combination of stiffness and adhesive properties could enhance chondrogenesis [71]. This suggests that other factors, such as adhesion combined with stiffness, will decide the terminal tendency of chondrogenic differentiation.

A series of representative genes were selected for validation with qRT-PCR in order to verify the sequencing results; genes included *ALP*, *RUNX2*, *TGFβ2*, *COL2A1*, *COL11A1*, *PPARγ*, *C/EBPα*, *FADS2*, *PK*, *CS*, *LDHA,* and *PFKM* (Figure 5E,F). Typically, the expression of *ALP* and *RUNX2* was slightly elevated with an increase in stiffness, which means that the potential for osteogenic differentiation was enhanced (Figure 5E). Consistently, the YAP/TAZ pathway has recently been recognized as a major regulator of cellular sensing and mechano-signaling transduction, enhancing nuclear translocation with increased ECM stiffness. The YAP/TAZ pathway could interact with, and activate, multiple DNA-binding partners, such as RUNX2 in the nucleus, and thus regulate the osteogenic differentiation of stem cells [72,73].

Furthermore, studies have reported that the induction of TGF-β contributes to the progression of ECM stiffness [74,75,76], which indicates that TGF-β is highly correlated with ECM stiffness. In this study, it was shown that with the up-regulation of stiffness, expression of *TGF-β* was elevated (Figure 5E). TGF-β plays a significant role in stimulation of osteogenesis of MSCs [77]. *Tgf-β1* knockout mice displayed reduction in bone growth and mineralization [78], while *Tgf-β2* and *Tgf-β3* double knockout mice had a deficiency of the distal rib [79]. Meanwhile, TGF-β signaling suppressed adipocyte maturation in MSCs [80]. In this study, we found that the up-regulation of *TGF-β2* expression level was accompanied by enhanced osteogenic capacity and reduced adipogenic capacity of MSCs. In chondrocytes, regulation of the TGF-β-related pathway is complicated and dose-dependent, with a number of aspects still remaining unclear. A low dose of TGF-β mainly activates downstream pathways of pSMAD2/3, whereas high doses activate pSMAD1/5 signaling pathways [81,82]. Notably, the chondrocyte pSMAD2/3 and pSMAD1/5 pathways have been revealed to be antagonistic toward each other. For example, the TGF-β-dependent pSMAD2/3 pathway inhibits hypertrophy and terminal differentiation of chondrocytes [82,83,84,85,86], whereas TGFβ-dependent pSMAD1/5 signaling is correlated with chondrocyte hypertrophy [81,82].

In contrast, the expression of *COL2A1* drastically decreased with increasing ECM stiffness (Figure 5E). The *COL2A1* gene encodes a type II collagen precursor, which is a well-known chondrogenic marker and is essential for cartilage development [87]. COL2A1 is involved in the regulation process of periosteal osteogenesis and endochondral osteogenesis. Mutation of COL2A1 causes structural abnormalities of type II collagen, resulting in a variety of osteochondral dysplasia diseases [88]. However, correlation of COL2A1 with adipocyte differentiation is still relatively unexplored.

COL11A1 is an important ECM molecule regulating collagen fibrillar assembly, which plays a pivotal role in endochondral ossification [89]. Functional knockout of *Col11a1* in mice resulted in defects in chondrogenesis, epiphyseal cartilage structure, and collagen fibers, suggesting COL11A1 plays a vital role in chondrogenic differentiation [90]. A recent study revealed that expression of *Col11a1* in the growth plate and perichondrium is important for newly formed bone during endochondral ossification [90]. Deletion of the *Col11a1* gene in developing bones of mice results in a thickened trabecular bone and reduces endosteal bone turnover [89]. In our study, we found that the expression of *COL11A1* was down-regulated with an increase in ECM stiffness, which was consistent with the trend of chondrogenic differentiation. One possible reason for this is that the microenvironment is simple and could not completely simulate the complicated natural bone structure.

PPARγ and C/EBPα are crucial regulators of adipocyte differentiation. The higher expression of *PPARγ* and *C/EBPα* in the soft group compared with the medium and stiff groups suggests that a soft ECM is beneficial for adipogenic differentiation (Figure 5E). FADS2 is a fatty acid desaturase that is involved in the regulation of lipid metabolism, and Fads2 knockout mice were found to exhibit impaired lipid synthesis [91]. In this study, the highest expression of *FADS2* was observed in the soft group with the highest adipogenic capacity. We also observed that with an increase in ECM stiffness, genes that encode the key enzymes of glycolysis and the TCA cycle, including *PK*, *CS*, *LDHA*, and *PFKM*, were gradually accumulated (Figure 5F). Fernie et al. [92] have shown that increased ECM stiffness promotes F-actin reorganization, leading to enhanced glycolysis.

Metabolites of glycolysis and TCA were down-regulated when cells were cultured on a soft ECM. Both PK and PFKM are glycolytic rate-limiting enzymes, which regulate the intracellular balance between energy and metabolism. A previous study showed that PFK is closely related to metabolic regulation in bone regeneration [93]. CS is a core enzyme of the TCA cycle, and it provides direct control of cellular function [94]. LDHA is an essential metabolic enzyme that catalyzes the process of converting pyruvate to lactate [95]. An in vivo study has found that the application of parathyroid hormone promotes bone regeneration, and it is accompanied by up-regulation of CS and LDHA levels in rats [96]. Another recent study showed that LDHA promotes osteoblast differentiation and the formation of mineralized nodules through histone lactylation [97]. According to the results, we could include the idea that ECM stiffness leads to metabolic reprogramming, which is also closely interrelated with the energy requirements of cell differentiation.

**Figure 6:**
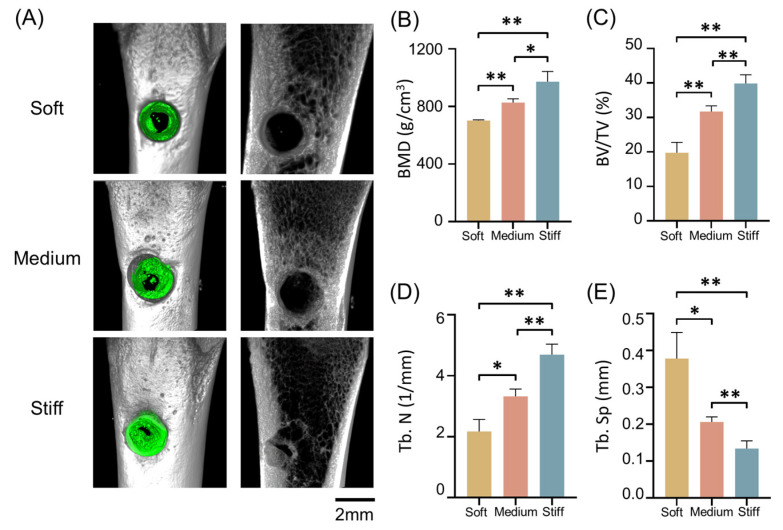
Investigation of the function of 3D ECM stiffness during bone healing in vivo. (**A**) Left line: 3D reconstruction of bone-like tissue (green) in soft, medium, and stiff groups. Right line: cross sections of the surgical area in soft, medium, and stiff groups. Scale bar is 2 mm. (**B**–**E**) Quantitative analysis of the (**B**) bone mineral density (BMD), (**C**) percentage of BV to TV (BV/TV) of bone tissue, (**D**) trabecular number (Tb.N), and (**E**) trabecular separation (Tb.Sp) of femurs with implantation of soft, medium, and stiff hydrogels. (* *p* < 0.05, ** *p* < 0.01, using one-way ANOVA).

Finally, to investigate the function of 3D ECM stiffness during bone healing, an in vivo rat model was used to evaluate the degree of bone healing at eight weeks. MSCs were implanted with GM30/60/90 into the distal femur. After eight weeks, the femurs were scanned using micro-CT. The micro-CT images showed that the most bone-like tissue formed within rats implanted with the stiff group, while in soft group, the least bone formation was observed (Figure 6A). The new bone-like tissue was evaluated and quantified using BMD, bone volume/total volume (BV/TV), trabecular number (Tb. N), and trabecular separation (Tb. Sp) (Figure 6B–E).

However, there are still some limitations in this research. Multi-omics research includes not only metabolomics and transcriptomics, but also genomics, epigenomics, and proteomics. For example, when the ECM stiffness changes, the proteins secreted by stem cells will be different. Proteomics can visually, clearly, and comprehensively detect the changes in secreted proteins and intracellular proteins. Epigenomics can specifically reveal the regulation mechanisms of gene expression and epigenetic modifications. In addition, the single-cell RNA-sequence technique (scRNA-seq) has been increasingly developed [98]. In the future, we will employ more advanced techniques, such as the single-cell RNA-sequence technique, to detect the precise effect of ECM stiffness on stem cell fate, thus providing new ideas for bone tissue engineering strategies.

## 3. Materials and Methods

### 3.1. Physicochemical Properties of GelMA

Compression modulus: Gelatin Methacryloyl (GelMA, EFL, Suzhou, China) is a kind of bio-hydrogel material with photosensitive properties. GelMA was obtained from Yongqinquan Intelligent Equipment Co., Ltd., Suzhou, China. The substitution rate of Methacryloyl is a core parameter for GelMA, which changes the forming and mechanical properties of materials. In our study, GM30/60/90 solutions with diverse substitution patterns were used to imitate different stiffnesses of ECMs. GM30/60/90 were mixed with a photoinitiator in certain proportions (GM30, 6%; GM60, 6%; GM90, 8%) [93]. To test the mechanical properties of GelMA, hydrogels of 2 mm thickness and 8 mm diameter were prepared. The compression modulus was determined using an electronic universal testing machine (Z020, ZwickRoell, Ulm, Germany). At a strain rate of 5 mm/min, the compression modulus of GelMA was measured at the first 10% slope of the stress/strain curve [99,100].

Fourier transform infrared (FTIR) spectroscopy test: The prepared GM30/60/90 matrices were dried and ground evenly with potassium bromide powder. Attenuated total reflection using Fourier transform infrared (ATR-FTIR) was used to confirm the chemical structure using a Bruker VERTEX80v FTIR spectrometer [93].

### 3.2. Cell Culture

BMMSCs (purchased from ScienCell Research Laboratory, Carlsbad, CA, USA) were passage 3, and they were cultured in GelMA at three stiffnesses (GM30/60/90) to simulate different ECM stiffnesses that influence BMMSCs’ fates. Cells in GelMA were cultured in Minimum Essential Medium α (α-MEM, Gibco, Grand Island, NY, USA), with addition of 10% fetal bovine serum (FBS, Gibco, Grand Island, NY, USA), 50 U/mL penicillin, and 50 μg/mL (Gibco, Grand Island, NY, USA) streptomycin in an incubator set at 37 °C, while maintaining 5% carbon dioxide. BMMSCs were blended with the GelMA solution with a final cell concentration of 2 × 10^6^/mL at 37 °C prior to cross-linking. The mixtures of cells and GelMA were added to the well plates, and they were light cured using 405 nm ultraviolet light for 90 s to encapsulate BMMSCs into the GelMA [93]. Then, the PM was added to the well plates.

### 3.3. Sequencing and Data Analysis

The extracted total RNA was sent to Beijing Cnkingbio Biotechnology Co., Ltd. (Beijing, China) for RNA sequencing (RNA-seq) [101]. BMMSCs in GM30/60/90 were sent to Lipidall Technologies Company Limited (Changzhou, China) for metabolic analysis, using ultra-high performance liquid chromatography (UHPLC) and tandem quadrupole time-of-flight mass spectrometry (QTOF-ms) [102]. Data analysis was also performed by Lipidall Technologies Company Limited. Non-targeted metabolism was defined as the quantitative detection of all endogenous metabolites with molecular weights less than 1000 daltons.

### 3.4. ALP Staining and Quantification

BMMSCs combined with GM30/60/90 were cultured in 48-well plates. Alkaline phosphatase (ALP) staining was performed on the 7th day of osteogenesis induction [103], using a 5-bromo-4-chloro-3-inodlyl-phosphate/nitroblue tetrazolium (BCIP/NBT) chromogenic kit (Beyotime, Shanghai, China). Cells were fixed in 95% absolute ethyl alcohol overnight before staining, and results were observed and recorded using an optical microscope. To detect ALP activity, a bicinchoninic acid (BCA) protein assay kit (Pierce Thermo Scientific, Waltham, MA, USA) and a quantitative assay kit for ALP (Nanjing Jiancheng Bioengineering, Nanjing, China) were used.

### 3.5. Oil Red O Staining and Quantification

The Oil Red O staining test was used to determine adipogenic differentiation of BMMSCs cultured in GM30/60/90 in 48-well plates on the 21st day of adipogenic induction [104,105]. Cells were fixed in 10% formalin neutral fixative for 1 h, and they were subsequently thrice rinsed with phosphate-buffered saline (PBS) followed by isopropanol. After being stained in fresh 3% Oil Red O solution for 30 min, cells were photographed using an optical microscope, and the percentage of cells staining positive was quantified (ImageJ 2.0.0).

### 3.6. Alcian Blue Staining and Quantification

3D cultured BMMSCs were placed in GM30/60/90 in 48-well plates in the same way. Cells were cultured with chondrocyte induction medium for 14 days, after which time they were stained using an Alcian blue staining kit (Sigma, Saint Louis, MO, USA). Cells were fixed in 4% paraformaldehyde for 1 h, and they were washed with PBS. Alcian blue solution was applied to cells for 30 min at room temperature and then washed off using PBS [106,107]. Cells were observed and photographed using the optical microscope. The percentage of stained positive cells was calculated (ImageJ 2.0.0).

### 3.7. Live/Dead Cell Staining

Cells in GM30/60/90 were treated with a Live/Dead cell kit (YEASEN, Shanghai, China) and then imaged using a Leica TCS SP8 confocal microscope. The 3D images were reconstructed to determine their viability [93].

### 3.8. Immunostaining of Cell Culture

Cells were cultured in GM30/60/90 for 7 days, fixed for 15 min in 4% araformaldehyde, and then permeabilized for 10 min in 0.1% (*v*/*v*) Triton X-100 at 4 °C. After washing cells with PBS, solutions of 4′,6-diamidino-2-phenylindole (DAPI) and anti-F-actin antibody were added, with cells exposed for 30 min at room temperature. Images were obtained using a Leica TCS SP8 confocal microscope [98].

### 3.9. qRT-PCR Analysis

After both 7 and 14 days of culture, cells were retrieved from the GM30/60/90 cultures for a real-time quantitative PCR (qRT-PCR) test. Gene expression of RUNX family transcription factor 2 (*RUNX2*), alkaline phosphatase (*ALP*), transforming growth factor beta 2 (*TGFβ2*)*,* collagen type II alpha 1 chain (*COL2A1*), collagen type XI alpha 1 chain (*COL11A1*)*,* peroxisome proliferator activated receptor γ (*PPARγ*)*,* CCAAT/enhancer binding protein α (*C/EBPα*), fatty acid desaturase 2 (*FADS2*), pyruvate kinase (*PK*), citrate synthase (*CS*), lactate dehydrogenase A (*LDHA*), and phosphofructokinase, muscle (*PFKM*) was determined. In brief, total RNA was reverse transcribed into cDNA using the PrimeScript RT kit (AG Scientific, Shanghai, China), and then the cDNA was used to perform qRT-PCR using SYBR Green Master Mix (YEASEN, China). GAPDH was regarded as the internal reference. The level of gene expression was measured using the 2^−ΔΔCt^ formula [93,99,101]. The forward and reverse primers are listed in Table 1.

### 3.10. Animal Model

The animal surgical experiments were approved by the Institutional Animal Care and Use Committee of Peking University (Study Number: LA2022239). A model relating critical size defects in the distal femoral bone of rats was derived. Eight-week-old male Sprague Dawley (SD) rats were anesthetized using 1% pentobarbital sodium. A longitudinal incision was taken on the lateral side of the distal femur to reveal the lateral femoral condyle, and the bone cortex was penetrated with a 2 mm drill. GM30/60/90 embedded with BMMSCs were implanted into the defect sites, and nothing was implanted in the control group. Each group included three rats, for a total of 12 rats. After 8 weeks, femurs were harvested and fixed in 10% neutral buffered formalin for 24 h. Micro-CT images of new bone formation within the bone defects were observed using an Inveon instrument (Siemens, Munich, Germany), and the images were then reconstructed using 3D visualization software (Inveon Research Workplace; Siemens, Munich, Germany) [93,106].

### 3.11. Statistical Analysis

Results were recorded as a mean ± standard error of the mean (SEM). Comparison between more than two groups was conducted using one-way ANOVA. Post hoc multiple comparisons between two groups used the LSD test [98,99]. *p* values of less than 0.05 were determined to be statistically significant. Statistical significance was defined as * *p* < 0.05, ** *p* < 0.01, and *** *p* < 0.001.

## 4. Conclusions

In summary, our extensive research has systematically mapped out the possible pathways through which ECM stiffness regulates stem cell fate; this provides a theoretical basis for designing smart bio-scaffolds for tissue engineering. These results offer deep insights into the role of ECM stiffness in altering cell metabolism and thus regulating stem cell differentiation. Moreover, this work connects metabolism and stem cell fate, providing potential therapeutic targets for metabolic diseases.

## Figures and Tables

**Table 1:** Primer sequence.

Primer	Forward (5′-3′)	Reverse (5′-3′)
*GAPDH*	GGTCACCAGGGCTGCTTTT	GGATCTCGCTCCTGGAAGATG
*ALP*	GACCTCCTCGGAAGACACTC	TGAAGGGCTTCTTGTCTGTG
*RUNX2*	CCGCCTCAGTGATTTAGGGC	GGGTCTGTAATCTGACTCTGTCC
*TGFB2*	CAACAGCACCAGGGACTTGC	AACTGGGCAGACAGTTTCGGA
*COL11A1*	TCCTGGACCACCAGGAAGGAT	GACCAGTCTCACCGGTTGGT
*COL2A1*	CCCATCTGCCCAACTGACCT	TTTGGTCCTGGTTGCCCACT
*PPARγ*	GAGGAGCCTAAGGTAAGGAG	GTCATTTCGTTAAAGGCTGA
*C/EBPα*	CGCAAGAGCCGAGATAAAGC	CACGGCTCAGCTGTTCCA
*FADS2*	TGACCGCAAGGTTTACAACAT	AGGCATCCGTTGCATCTTCTC
*PK*	AGAGAGGCAGCCTTCAGACCT	CTGTTTTGTGCCCCGCAAGA
*CS*	TGGGTGTACTGGCACAGCTC	GTGCTCATGGACTTGGGCCT
*LDHA*	GGCTTGAGCTTTGTGGCAGT	GGCTCCTACAGCAAGGACACA
*PFKM*	GAGCACCATGCAGCCAAAAC	GCAGCATTCATACCTTGGGC

## Data Availability

Data is available on request.

## Supplementary Materials

The following supporting information can be downloaded at: https://www.mdpi.com/article/10.3390/ijms24119311/s1.
