# Supplementary material for: Integrative Analysis Reveals the Diverse Effects of 3D Stiffness upon Stem Cell Fate
Source: Int J Mol Sci. 2023 May 26;24(11):9311. doi: 10.3390/ijms24119311 (PMC10253631; doi:10.3390/ijms24119311)
Supplement: Supplementary file 1 [file ijms-24-09311-s001.zip › ijms-2389013-supplementary.pdf]

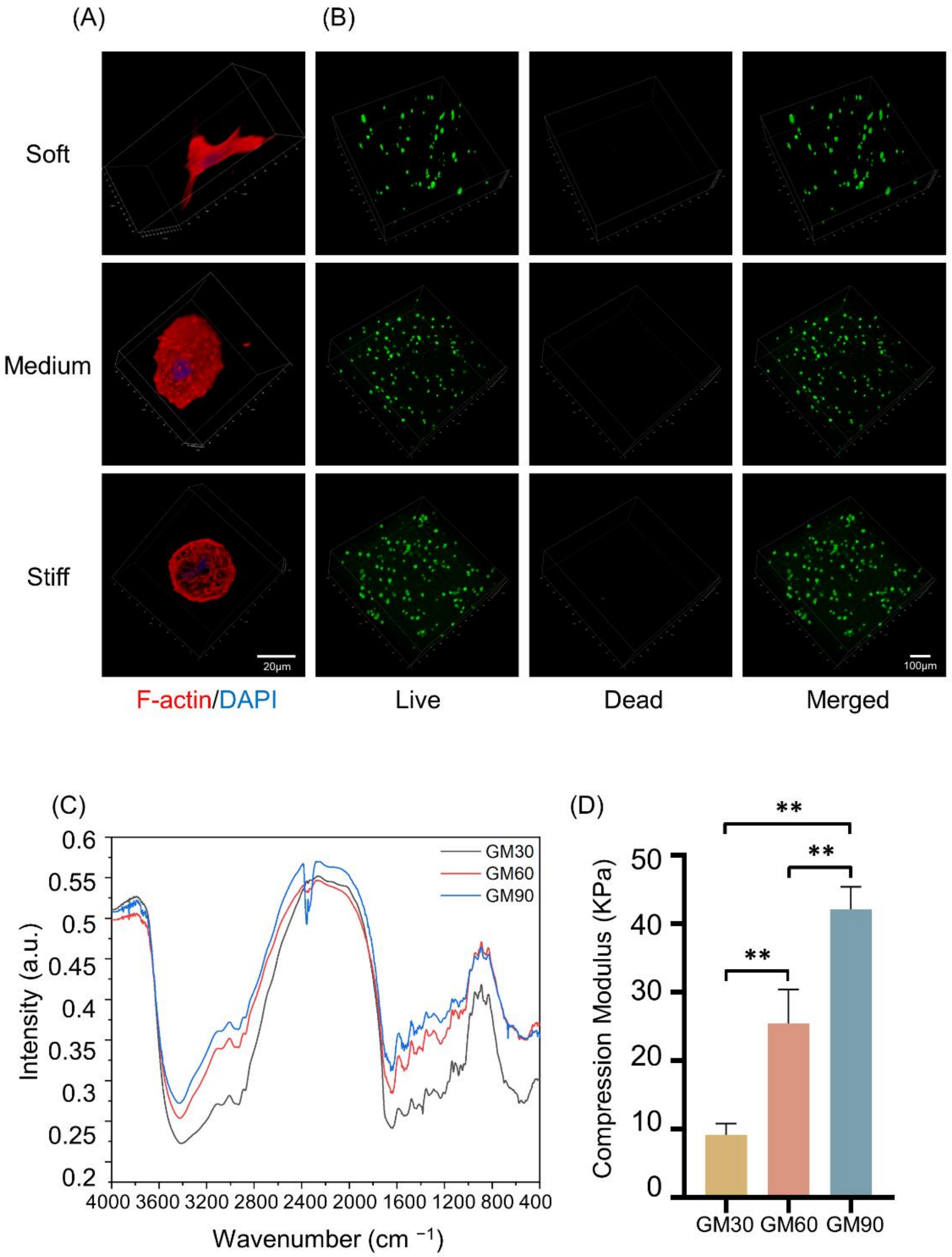

Supplementary Figure S1. Characterization of GelMA. (A) Immunofluorescence showed that with the increased stiffness of ECM, the cells gradually became smaller and rounder, while the cells in the soft ECM became elongated. Scale bar is 20 μm. (B) Live/dead staining showed that cells cultured in soft, medium and stiff groups showed good viability. Scale bar is 100 μm. (C) Fourier-transform infrared (FTIR) spectroscopy test. (D) Compression modulus of three kinds of GelMA with different stiffness (***P* < 0.01, by one-way ANOVA).

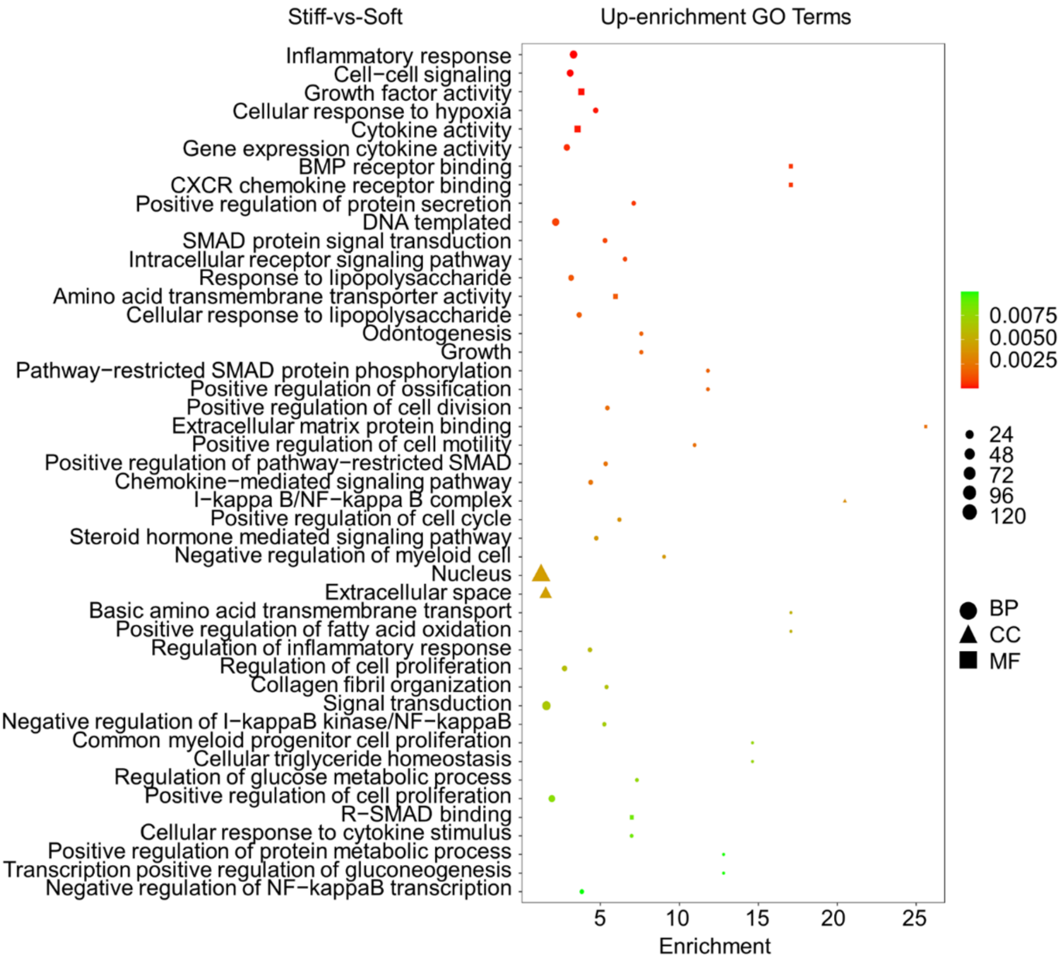

Supplementary Figure S2. GO enrichment analysis of up-regulated genes in the stiff-vs-soft group.

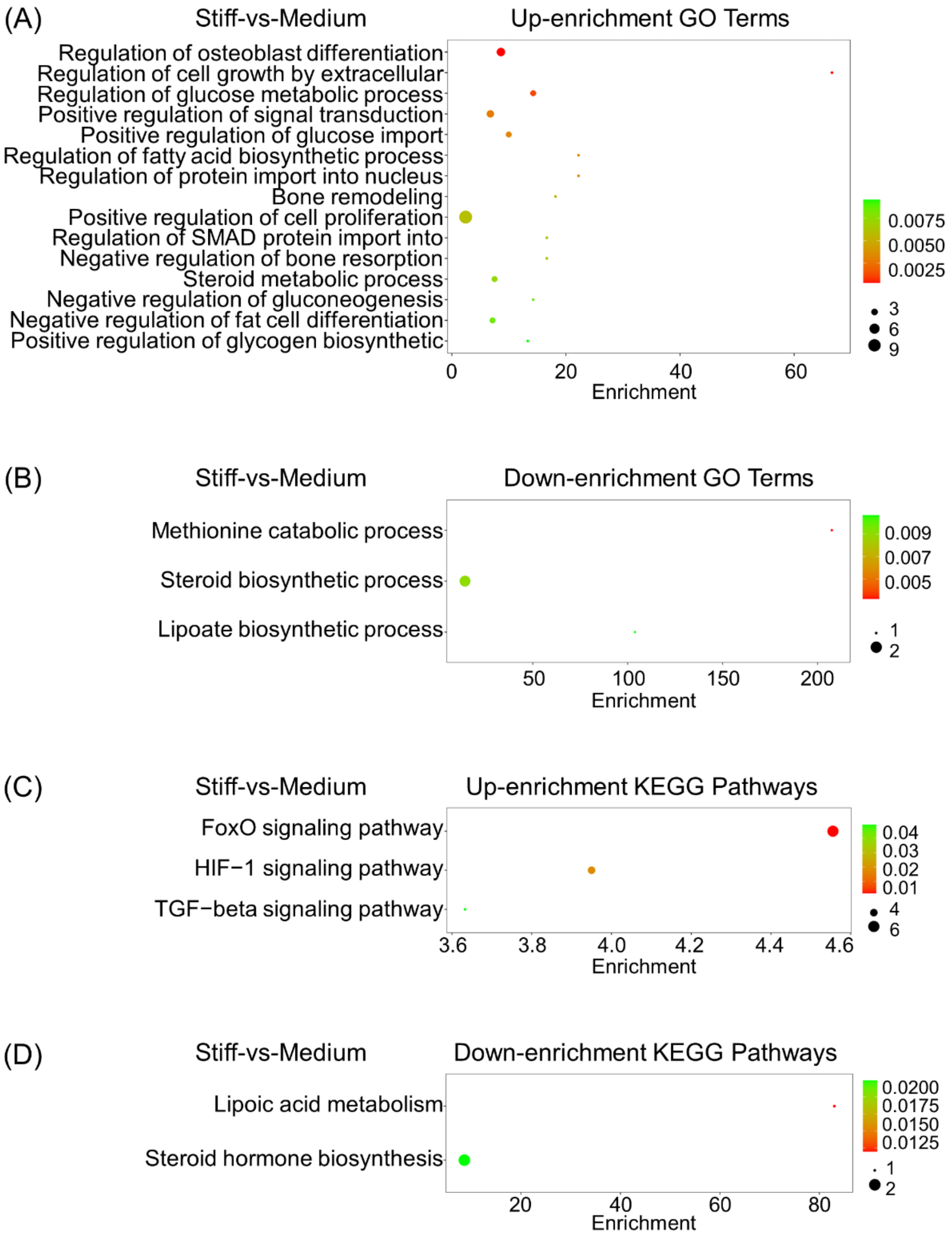

Supplementary Figure S3. GO and KEGG pathway analysis of DEGs in the stiff-vs-medium group. (A) Up-regulated genes (B) Down-regulated genes in GO enrichment analysis between stiff and medium group. (C) KEGG pathway analysis of up-regulated genes in the stiff-vs-medium group. (D) KEGG pathway analysis of down-regulated genes in the stiff-vs-medium group.

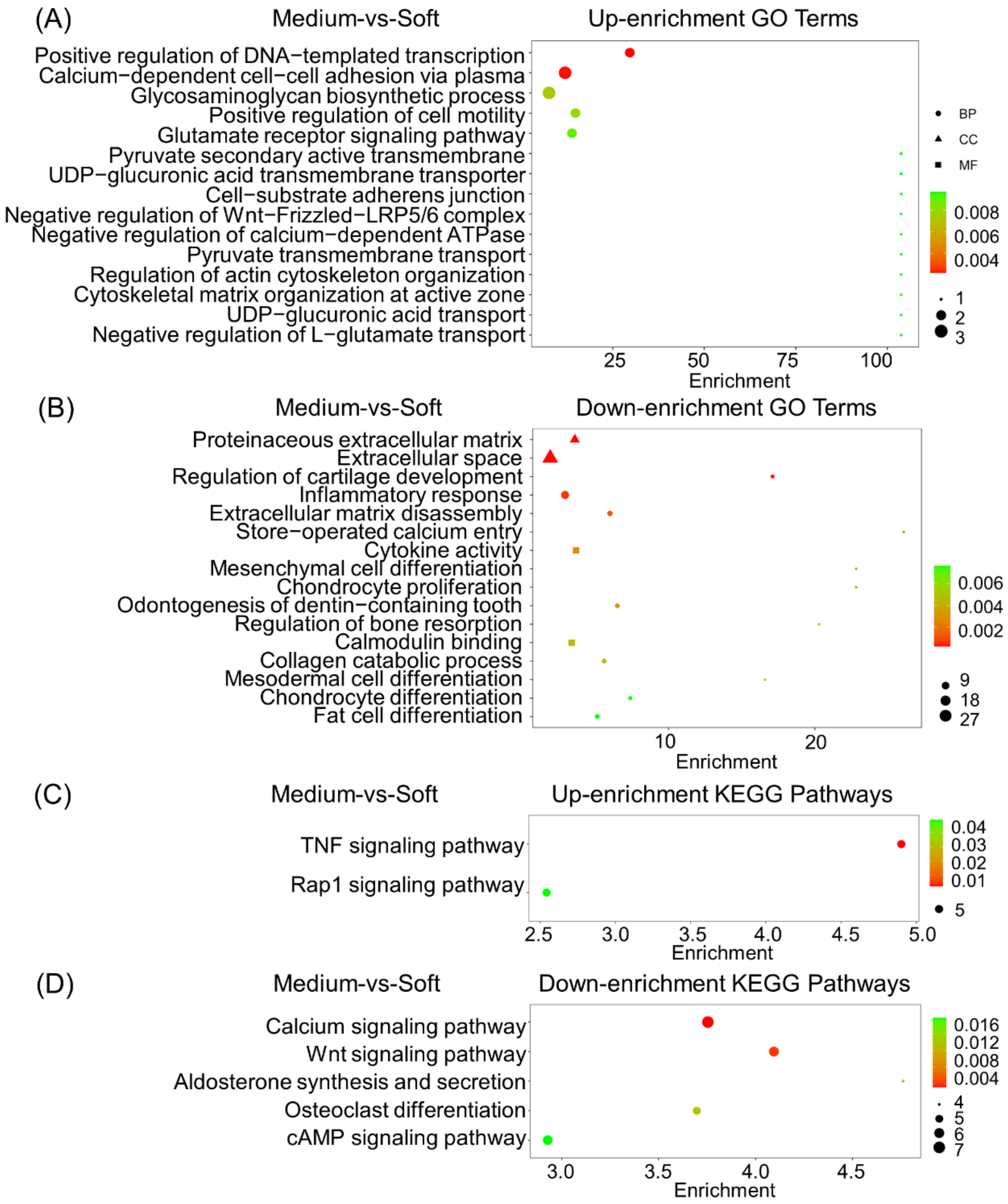

Supplementary Figure S4. GO and KEGG pathway analysis of DEGs in the medium-vs-soft group. (A) Up-regulated genes (B) Down-regulated genes in GO enrichment analysis between medium and soft group. (C) KEGG pathway analysis of up-regulated genes in the medium-vs-soft group. (D) KEGG pathway analysis of down-regulated genes in the medium-vs-soft group.

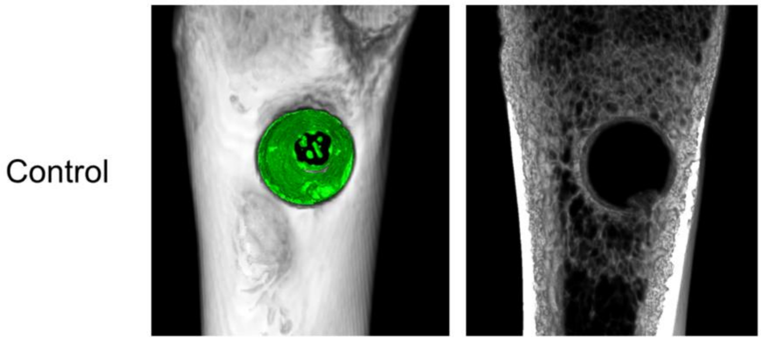

Supplementary Figure S5. 3D reconstruction of bone-like tissue (green) in control group.
